# Informal support for people with Alzheimer’s disease and related dementias in rural Uganda: a qualitative study

**DOI:** 10.1186/s13033-020-00364-9

**Published:** 2020-05-07

**Authors:** Pia Ngoma Nankinga, Samuel Maling, Zeina Chemali, Edith K. Wakida, Celestino Obua, Elialilia S. Okello

**Affiliations:** 1 Medical Simulation Center, Mbarara University of Science and Technology, Mbarara, Uganda; 2 Department of Psychiatry, Faculty of Medicine, Mbarara University of Science and Technology, Mbarara, Uganda; 3 Departments of Neurology and Psychiatry, Harvard Medical School, Massachusetts General Hospital, Boston, MA USA; 4 Office of Research Administration, Mbarara University of Science and Technology, Mbarara, Uganda; 5 Department of Pharmacology and Therapeutics, Mbarara University of Science and Technology, Mbarara, Uganda; 6 Mwanza Intervention Trials Unit, Mwanza, Tanzania

**Keywords:** Informal support, Dementia and rural communities

## Abstract

**Background:**

The generation of people getting older has become a public health concern worldwide. People aged 65 and above are the most at risk for Alzheimer’s disease which is associated with physical and behavioral changes. This nurtures informal support needs for people living with dementia where their families together with other community members are the core providers of day to day care for them in the rural setting. Despite global concern around this issue, information is still lacking on informal support delivered to these people with dementia.

**Objective:**

Our study aimed at establishing the nature of informal support provided for people with dementia (PWDs) and its perceived usefulness in rural communities in South Western Uganda.

**Methods:**

This was a qualitative study that adopted a descriptive design and conducted among 22 caregivers and 8 opinion leaders in rural communities of Kabale, Mbarara and Ibanda districts in South Western Uganda. The study included dementia caregivers who had been in that role for a period of at least 6 months and opinion leaders in the community. We excluded trained health workers.

**Results:**

The study highlights important forms of informal support offered to PWDs such as support in activities of daily living, enabling access to medical attention, recovering misplaced items, provision of herbal remedy, informal counseling, and sourcing carers from other families to offer presence and support in the hope to impact positively on behavioral outbursts and the frustration of living with dementia.

**Conclusion:**

The study revealed various forms of informal support that are available for PWDs in South Western Uganda and stressed the role of caregivers and the perceived usefulness of the care provided.

## Introduction

Population ageing has become a global concern [1]. The ageing of the population is expected to lead to an increase in the prevalence of dementia [2]. Despite advances in health-based remedies to control symptoms and stretch the disease trajectory, the managing of dementia in Africa still largely consists of sheltered care throughout a sustained trajectory of decline [3]. According to the Uganda Bureau Of Statistics 2016 report, out of the 212,446 elderly of 60 years and above in Southwestern Uganda, 90,039 are men and 122,407 are women [4]. A recent study indicated that 20% of the population of those aged 60 years and over in Southwestern Uganda have dementia implying a relatively high prevalence of dementia [5]. Much of the care for persons with chronic illnesses and disability is provided by their families and other informal structures within the community [6]. Alzheimer’s disease is the most common type of dementia and accounts for 60–70% of cases [7]. Early in the disease with insight still intact, it can be very frustrating for the person with Alzheimer’s disease or other dementias as they experience changes in their abilities. Feelings of losing control and increased frustration combined with the physical changes of aging may cause the person to have low frustration tolerance with emotional outbursts [8].

Mental health services have not been fully established and recognized by the general inhabitants in developing countries and therefore a pronounced number of people with dementia and the caregivers utilize other options of services such as psychosocial and spiritual support from families and communities [9]. The socio-cultural environment or view of dementia care in Sub-Saharan African locales is influenced by the customs and attitude of people including those taking care of people with dementia. In numerous parts of Nigeria for example, indicators of hallucination and delusion in the perspective of dementia may be interpreted as spiritual. Such beliefs together with the prevailing intergenerational living arrangement in many African societies averts a big number of people with dementia from presenting for treatment at health care facilities and families resort to taking care of the people with dementia in their homes until their behavior becomes too difficult to cope [10].

According to Omugurusi Festo Karwemera’s narration on Voice of Kigezi in 2015 on the program “Kankuganire” about the care of people with dementia in Uganda, Kigezi culture. Karwemera states that “okyizongozya abanyu warutsya, abagurusi bashandaga obutaka, ohumura. Oyeba nkekirooto, ohingura”. This translates into “after shaking out all your people’s effort to take care of you, the old men cut the soil and you rest”. This means that the caregivers and family members of people with dementia that provided informal support to the elders found it burdensome and time-consuming and as the person grew older, they found ways of gradually watching them to their last breath. This is similar to the historical norm among the Karamojong ethnic group in the North east of Uganda whose main livelihood activity was herding livestock. The younger family members took care of the elderly inclusive of those with dementia in their homes. At a later stage, the younger family members left the elderly in the homesteads as they left for cattle grazing and never returned to the home and moved far away where they settled in new homes. This kept the elderly isolated as they gradually met their demise.

This explains the African historical practice when taking care of people with dementia. This practice resorting to get rid of the pressing needs of an aging person stems from the lack of education and training in basic skills on how to care for people with dementia in the rural communities of south western Uganda. Based on this culture, the aged including those with dementia, were given home care by the young ones in the family who provided them direct support in the family and lived with them at all times. With new changes in the socio-economic patterns and with children leaving to urban settings to earn a living, the elderly and PWDs are left in the care of the sons and daughters-in-law and grandchildren who may not provide adequate care for many reasons [11]. With that background, our study set out to explore the nature of informal support provided to people with dementia in rural communities in Southwestern Uganda.

## Method

### Study population

The research was conducted in three districts in southwestern Uganda. Study participants were dementia caregivers and opinion leaders in the community. In this study, a caregiver was defined as a family member or relative who rendered assistance to a PWD while living with them and had been in that role for a period of at least 6 months. Given our study’s focus, opinion leaders were selected because they are indirectly involved in supporting people with dementia and their opinions are highly valued in the community.

The study was conducted in Mbarara, Kabale and Ibanda districts. The study involved 30 participants, 22 were caregivers and 8 were opinion leaders. 11 of them were male and 19 were female who ranged between the age of 20 and 78 years. Opinion leaders were catechists, local councilors, herbalists, and a pastor. 28 participants practiced farming for a living, one was a constructor and one was a professional teacher. The study excluded trained health workers in the community and individuals younger than 18 years of age.

### Study design and sampling procedure

The study was community based and adopted a qualitative descriptive design. In the first stage, three districts were selected purposively out of the sixteen districts in southwestern Uganda as Mbarara and Kabale have the highest population in Southwestern Uganda. According to Wikipedia, Kabale has a population of 528,231 and Mbarara has 472,629 people as of the 2014 population census and this is high as compared to other districts in south western Uganda. In the second stage, one sub-county was randomly selected from a list of all sub-counties in each of the three districts. In the third stage, one parish was randomly selected from each of the selected sub-counties. In each parish, we went door to door recruiting caregivers using lists of families of people suspected to have dementia and opinion leaders in the community and these lists were provided by the Village Health Team members. All consented participants accepted to participate in the study. The point of saturation in the parish determined sample size.

### Data collection tools

The study used interview guides. These were developed basing on the customs and culture in taking care of the elderly especially those with dementia in the rural setting. The questions in the interview guide were also based on literature. Data was collected using an audio recorder to capture the respondents’ answers. Some notes were written down to back up the audios.

### Data collection procedure

The research team used gatekeepers from the community such as local leaders who introduced the research team to Village Health Team members (VHTs). The VHTs took the research team to homes where the people suspected to have dementia lived. Caregivers of elders confirmed to have memory loss, screened using the Brief Community Screening Interview for Dementia (CSI-D), consented with written consent and the interview guide was administered. The data from caregivers was collected using an in depth interview where responses of participants were recorded using an audio recorder and written notes to back up the recorded data. The interview guide contained questions assessing informal support provided and the perceived usefulness of the informal support provided to people with dementia. Opinion leaders defined as people whose ideas are highly valued in the community were also consented and interviewed individually using a key informant guide. The interview guides were both in English and Runyankole (see Appendices 1, 2, 3 & 4).

### Data analysis

Our aim was to establish the nature of informal support provided for people with dementia (PWDs) and the perceived usefulness of this support in rural communities in South Western Uganda.

Data collected from interviews was translated and transcribed. The transcription was checked against the audio for mistakes and ready for analysis. Data was grouped matching the research questions with the respondents’ replies and analyzed using thematic analysis approach. Overarching themes emerged from various collected sub themes specifically related to respondents’ answers.

### Quality control

Researchers were paired. This allowed one researcher to lead the interview and record while the other researcher was taking additional notes and charting comments. Research assistants for this study were graduates in social sciences (community planning and development) and were experienced in data collection. They were trained on the design tools with the satisfactory conduction of the interview guides.

At the end of each day of data collection, there was a review with the research team to check for the completeness of the interviews. The design tools were translated into the local language (Runyankole-Rukiga) to cater for those who preferred to be interviewed in those languages. Audio recording tapes were checked before use. The research team listened to the tapes. The research team got feedback with evaluation and monitoring on a daily basis to ensure high quality field research.

### Ethical consideration

An administrative clearance was obtained from the Chief Administrative Offices of each district where the study was conducted, allowing the research team to carry out the research in the communities. The Research Ethics Committee of Mbarara University of Science and Technology and the Uganda National Council of Science and Technology (clearance number SS 4852) approved the study. Informed consent was obtained from participants and consent for people with dementia who could not provide informed consent due to cognitive difficulties, informed consent to participate in screening was obtained from their caregivers who assented both verbally and in writing. PWDs that forgot responses to consent were known to have low capability to consent and thus their caregivers provided assent. Codes were used instead of using the participants’ names as a way of maintaining confidentiality. Participants were assured private spaces to meet for the interview and to record their responses.

## Results

### Socio-demographic characteristics of participants

The study involved 30 participants, 22 were caregivers and 8 were opinion leaders.

The participants’ ages ranged between 20 and 78 years, with 11 males and 19 females. Opinion leaders were catechists, local councilors, herbalists, and a pastor. 28 participants practiced farming for a living, one was a builder and one was a teacher.

### Study findings

Two major themes emerged: (1) Informal support were available to PWDs and (2) The perceived usefulness of the support.

### Theme 1: Informal support provided in the homes

This theme emerged from eleven categories namely, supporting and reminding of activities of daily living, reminding PWDs to attend social and family activities, creating a socially connected environment, enabling accessibility to medical care, assisting with occupational activities, recovering misplaced items, informal counseling and guidance, spiritual nourishing, providing of herbal remedy, looking for financial support and sourcing carers from other families.

### Supporting and reminding of activities of daily living

Caregivers explained that they supported PWDs by reminding them of the day to day activities. Most of the participants talked about cooking for them, bathing them, reminding them to take their medicine, washing their clothing and dressing. This was evidenced in the statements below;

“I bring for him food to eat, bring him a jug of water to use for bathing and also collect his clothing for washing. I have to keep him safe and also remind him of something should he forget.”

“I help fasten buttons for him or if he wears a shirt inside-out, I help him wear it the right way.”

“I’m the one who cooks for him… I do his laundry, lay his bed and also cook for him food. Things like that.”

And also helped them to search for misplaced items leading at time the caregivers to donate the replacement for the lost items. The caregivers also described that they reminded the PWDs to stay away from the sun as PWDs sat outside and forgot to move to shaded areas for protection from noxious sun rays and dehydration. This is evident in the statement below;

“..I remind her when she forgets, when she misplaces something I tell her to take her time to search for it this helps her to find what she is looking for and even help her search for it or even at times I give her my own to stuff to use if she totally fails to get hers.”

### Reminding them to attend social and family activities

Participants explained that they reminded PWDs to attend social activities since they forgot to do so. Whenever there was a social activity and gathering, the caregivers would go with PWDs and attend with them burials of deceased relatives, wedding parties, church services, going to the market as they forgot the market days. This was expressed as below;

“…if there is a party amongst the family. I tell him that today is the day of the party and he should go attend it…”

“Another thing that I usually do for him is reminding him on the days of the market…”

“.. She usually forgets by the next morning what was agreed on a day before…especially the days of the savings groups. I usually remind her of those days when meetings are held. She can never remember them…”

### Creating a less isolated environment

The caregivers talked about keeping company for PWDs to decrease their isolation and the feeling of not being loved. The caregivers comforted them during that time because PWDs thought they were bewitched. Moreover, caregivers ensured that there were batteries in the radio as they often used radio broadcasting for prayers since churches were not easily accessible for PWDs. This is evidenced as below:

“..as for church he no longer goes there that he has backache however he is always aware of Sundays because he listens to Radio Maria. He prays from the radio…”

“I normally buy him batteries that he uses to listen to the radio, buy…”

### Enabling accessibility of medical attention

The caregivers also explained that they assisted the PWD to easily access medical attention by inviting health workers home whenever the PWDs were unable to go outside for their medical visits as well as escorting PWDs to the hospital and keeping their medical forms for easy reference to their medical history. Caregivers also helped reminding PWDs to take their medicine as quoted below:

“..I also give her company by telling stories with her and also make sure she gets treatment when she is in need…”

The participants also shared the experience of these people with having lost their way and end up taking different routes such as going to the hospital for medication and they divert. These are evidenced as below;

“…For instance today I had to escort her to the health center because I knew if she went alone she would not make it and she would even disturb the health workers.”

“…I always tell her how the drugs were prescribed to her. I remind her of the different schedules she is supposed to take them.”

The statements collected further showed that the caregivers invited health workers for to the homes of PWD for medical examinations when the PWDs were unable to go to hospital.

The caregivers also explained that the children of PWDs took the PWDs whenever they were sick. The caregivers of these people notified the children who assisted in the transfer to the hospital for treatment. The caregivers also explained that they kept and supervised PWDs on taking medicine whenever they were sick to avoid mixing up the medications or taking accidental overdose.

“…I always tell her how the drugs were prescribed to her. I remind her of the different schedules she is supposed to take them.”

### Assisting in occupational activities

The caregivers described that PWDs could no longer carry themselves to the gardens or to the farms thus their caregivers assisted in taking the items of the PWDS to the market, growing food for them, harvested and left some harvest to feed on as evidenced in the following quote:

“….he has land but he doesn’t remember all of them. I am the one who goes there and takes care of the land so that (baby making noise) I grow crops and we get what to eat.”

### Recovering their misplaced items

Caregivers explained that they assisted the PWDs recover the misplaced items due to forgetfulness as evidenced:

“.. when she misplaces something I tell her to take her time to search for it this helps her to find what she is looking for and even help her search for it or even at times I give her my own to stuff to use if she totally fails to get hers.”

They further explained that the grandchildren and neighbors also helped them search for the misplaced items

### Informal counseling and guidance

Church leaders explained that they kept an eye on PWDs in places of worship as they tended to act contrary to the scheduled programs during worship and they offered them counseling both in their homes and at church. This was evidenced as below:

“..I usually offer them counseling because I notice them in their situation.”

They further explained that charismatic committees were in place and groups were trained on how to counsel and guide the PWDs as quoted below:

“..we have charismatic at church where we are usually trained on counselling so when someone tells you about such a situation we try to counsel them so that they feel at home…”

The church leaders also explained that there were ushering groups that assisted the PWDs whenever they went astray. These designated groups assisted them during prayers to keep orderly as expressed below:

“..in our church we have ushers who always are on the look of making sure there is order in church and they usually help such aged people with dementia whenever they are confused and acting on the contrary.”

### Spiritual nourishing

The caregivers pointed out that that the church leaders provided spiritual nourishing as they visited PWDs to pray for them, preaching about their problem and bringing the holy Eucharist to their homes when they couldn’t make it to church for prayers as evidenced below:

“…. those ones do. The Charismatic usually comes to pray and pray for him. The religious leaders also offer him the Eucharist here at home. If he has enough energy, he gets to go to the church and pray.”

### Providing herbal remedy

The traditional healers and general herbalists explained that they provided the PWDs with specific herbs to curb dementia. They stated that some herbs were used as salt baths and others were ingested. The traditional healers explained that before the herbs were given to the PWDs, words are whispered when the potion is mixed for the PWD to use as expressed below:

“….I look for medicine that I give them and the other one is for bathing. That’s my secret, when I prepare for them medicine it is up to me to tell you how to use it…. because there are some words I have to mention towards the medicine I give them.”

### Finding financial support

The caregivers identified children and grandchildren of the PWDs as their financial supporters. Most of them stated that PWDs’ children always came to visit and left them with some money to buy basic needs and brought items such as bread, sugar, salt and soap as expressed below:

“…. she has her brother who usually comes to check on her. He usually brings her some beans when it’s the season or leaves her with some money to buy soap.”

### Sourcing carers from other families

The caregivers described that younger relatives, grandchildren and daughters-in law of the PWD, were sent to stay at home. The grandchildren also held conversations with the PWDs in their free time while at their homes so the PWDs did not feel isolated and less valued. These were evident in the following quotes:

“..I also give her company by telling stories with her…. “

“…for example his sons and grandchildren… sending them for a plate in the house they may bring it especially (points at the grandson) that boy who stays with him in the house.”

### Theme 2: Perceived usefulness of the support

In relation to this theme, the categories included happiness and feeling loved, fulfillment, peace of mind, as well as relief and occupation.

### Happiness and feeling loved

The caregivers stated that whenever children of the PWDs visited them and supported them financially, PWDs felt happy, loved and cared for even when the children were not staying with them as one exclaimed;

“I see him become happy for example when he sends him money for treatment he comes back smiling and happy saying “my son has remembered me.”…..”

“He becomes happy when he sees people look out for him, he doesn’t get to suffer…”

### Fulfillment

Caregivers described that whenever they provided PWDs with daily support in cooking for them, helping them dress appropriately, washing their clothing and preparing for them water for bathing among others, PWDs expressed fulfillment and they did not have to struggle with the undone and forgotten house chores

This is evident in the quote below:

“He doesn’t suffer with cooking or tilling the land because we are the ones that do it for him…”

*“…*he feels great in his life like when someone grows crops for you because you’re unable to do so you feel good so he also feels good if he needs something and they bring it, life remains good…”

A church leader explained that the PWDs expressed satisfaction as they stopped worrying. He also pointed out that they also guided their caregivers oh how to assist them in their homes and this counseling was helpful and yielded as evidenced in the quote below;

“…it always takes time for such people to come back for assistance after offering them counselling; so that helps.”

### Peace of mind

Caregivers pointed out that when they held conversations and shared experiences PWDs had peace of mind with less worry given that they had people they counted on in the home as evidenced below:

“….as you know when someone grows old, she behaves like a child eeh! So sometimes she appreciates (Laughs a bit). I kill boredom at home, I give her company, we tell both old and new stories and this makes her feel that she has people at home.”

### Relief

Caregivers stated that PWDs were relieved of the hustle of endlessly looking for their misplaced items and not finding them. Having grandchildren and caregivers search for the misplaced items was always a point of relief as evidenced below:

“..it helps her in a way that for instance when she is looking for something and they tell her where it is she is relieved from the hurdle of searching everywhere.”

“….she becomes happy because she is aged and this helps her get back to her normal moods and it relieves her.”

Herbalists also explained that they gave PWDs herbs medicine for dementia and they were relieved since there was a subjective perception that they were healed from forgetfulness. This was evidenced in the quote below:

“There are those you can give herbs now, they’ll take them and become okay after a short while and if they continue to use them, they are healed.”

### Occupation

Caregivers explained that they kept the PWDs busy by turning on the radios when they were away and for those that could not make it to church they also listened to radio Maria and followed prayers on the radio. This is evident in quote below:

“I normally buy him batteries that he uses to listen to the radio…”

The caregivers also explained that they went with the PWDs to the garden to assist them and in so doing kept them busy rather than left at home to think about anything. They said that it kept the PWDs occupied as stated below;

“..In his condition, when he becomes busy with the farming, he will be occupied. He won’t have time to think about things that will disturb his peace of mind but rather focus on his garden. If we don’t give him that, he gets uncomfortable when he’s here.”

## Discussion

This study shows that the informal support available to PWDs in southwestern Uganda include supporting and reminding of activities of daily living, reminding PWDs to attend social and family activities, creating a less isolated environment, enabling accessibility of medical attention, assisting in occupational activities, recovering misplaced items, informal counseling and guidance, spiritual nourishing, providing herbal remedy, finding financial support, sourcing carers from other families and enhancing occupation and structured days. It also highlighted the perceived usefulness of those services to provide happiness and feeling loved, fulfillment, peace of mind, relief and offering a meaningful occupation.

### Theme 1: Informal support provided in the homes

This study shows that PWDs are vulnerable because of their forgetfulness and require extra support for good quality of life. This agrees with Gad Marshall who clearly explains the forgetfulness that is associated with dementia and how it leads to lack of autonomy and dependency on others for activities of daily living. Moreover, close family members are much more concerned than the person is about incidents of memory loss while the affected person loses interest in social activities and/or exhibits socially inappropriate behaviors [12].

This study shows that caregivers provide support in activities of daily living, feeding the PWDs, bathing them, washing their clothing. Similar findings were reported on informal services for taking care and ensuring the wellbeing of the PWDs is not compromised and ensuring the PWDs live as they would had they not suffered this condition [13].

As people age and consequently get affected by dementia, they tend to feel neglected and not loved. In this study, the caregivers of PWDs tried their best to keep these people from feeling isolated and lonely as they sent younger relatives to stay with them. Similarly, there was provision of company who stayed with PWDs at home made them feel less lonely [13]. In addition, support groups are found to be helpful to the families where PWDs live as they increase social support through visiting them and sharing experiences in the event of socializing. This creates a less isolated environment thus supporting PWDs live better [13].

In agreement with Guy Hindley’s findings, the caregivers in our study provided company to the PWD and decreased the perception of feeling isolated and not loved. Some of PWDs were very scared and panicked when left alone since the perception is that their condition is because of witchcraft. “*She was hallucinating and seeing snakes and dogs and things that don’t exist… and she said that someone held her head when she attended the wedding and the problems started ever since. Maybe the person who held her [head] had some bad powers and since that day her problems have started… We had to try treating her by taking her to FHs first, because this person who did this to her wanted Grandmother not to work at all and to be dependent… [On visiting the FH she] fell on the floor, and we were told to pray for her, because she had lost her memory. That’s when the devil left her and got back in, I think, and the healers kept on saying ‘get out, get out! This is Jesus’ blood’… They told us that we should continue praying for her when she goes to bed. If it wasn’t for this, her condition would have been worse, she wasn’t eating at all”* [14]. This accompaniment has enabled them to live longer and healthier.

Gad reported that medical attention is an integral support to PWDs as many may need several medications to treat aggression, agitation, depression and anxiety [12]. In our study, the care givers enabled the PWD to access medical care by inviting health workers home whenever the PWDs needed this support. The study further reveals that caregivers escorted PWDs to obtain medical assistance, kept their paperwork in check and reminded them to take their medication and kept the medicine for safe administration.

This study stresses that PWDs were supported in carrying out their occupational activities like farming. The Alzheimer Australia Report stresses need to assist the people with dementia in their day-to-day living, occupational activities and service access. Some of the activities highlighted in this report were access to appropriate community services, including personal care and domestic services as well as access to activities for PWDs and their caregivers, and satisfaction with service provision [15]. Support given by caregivers to PWDs in our study was in agreement with the board findings which recommend provision of emotional and social support. The report recommends counseling services as a strategy provided by the locals. This would effectively manage the challenging aspects of dementia as these locals are well acquainted with the day-to-day living of the dementia patients. Their services would be tailored and customized towards the relevant challenges affecting the people [16]. The report calls for backing this strategy with education and awareness needs which include knowledge and understanding of dementia for carers and the local counselors, and training them to be professionals [15]. We found that the counseling and guidance given by church leaders in southwestern Uganda is the first step in that direction, knowing though that more training and education is needed to fill the gap. In addition, the Anger/Frustration and Dementia fact sheet stresses the need to consider talking to someone who shares the experience and understands thus encourages counseling [8]. This is similar to the findings in our study as church groups compassionately supported PWDs in their own communities, understanding the challenges and frustration associated with the disease.

In this study, results show that church leader visited PWD, prayed for them as sometimes they came in church groups to provide spiritual support in the PWDs homes after identifying that they had abandoned the days for prayers and spiritual gatherings. Traditional healers and Christian faith healers have a big role in identifying PWDs as they first visit traditional healers and church faith healers to seek answers and remedies. [14]. Uwakwe also identifies the practice of assistance through finances and materials to the elderly members of the church with dementia seemingly the highest point of care for dementia patients in Nigeria [9]. This is in conformity with the findings in our study as the church brought items to support people with dementia in their homes through continuous visits.

Hindley’s findings stress the fact that several people with dementia reported that faith healers find them in their places of residence to pray for them for a general improvement in their health [14]. They added that traditional healers and herbalists are a category of informal support providers to dementia patients. In their study they reveal reasons why people with dementia visit traditional healers for support, as some think they are bewitched and some deem it ill health. In their study they reveal that people with dementia and their carers who visited their traditional healers would receive courses of one to two weeks of herbs provided and how to use them. And it is reported that people with dementia were believed to be bewitched and experienced slight improvements in their memory. They don’t show indications that these healers were diagnosing or treating a specific problem but rather simply praying for PWDs and telling them to read the Bible. Our results from southwestern Uganda provinces pinpoint that this type of informal support to PWDs was undertaken by church groups and leaders visited them in their homes for prayers with the PWDs.

In our study relatives of the PWDs always came to visit, offered care and supported them financially even while away. In fact, family members were always sourced to provide care to the PWD. The grandchildren, sons and daughters always stepped into provide support. According to Orpin, these caregivers are considered as primary carers who provide much of the day to day care for community dwelling people living with dementia. In his study he stresses that for the caregivers’ contribution to be effective they must be facilitated to have a deep understanding of their role [13]. The Alzheimer’s disease International Annual Report 2007–8 shows that the caregivers of PWDs maintained closeness with their families in supporting the PWDs [17].

Lastly, PWDs expressed fulfillment for the support rendered to them such as helping them with the activities of daily living, washing their clothes, accessing medical care etc. Fulfillment was also expressed for the support rendered to them by the herbalists. Hindley reported similar findings while studying PWDs in Tanzania. And it is reported that people with dementia who believed to be bewitched experienced slight improvements in their memory. This is in conformity with our study’s findings of herbalists providing herbal medicines and reporting PWDs to experience some recovery from their forgetfulness.

## Limitations

This study was a qualitative research and did not cover the entire South Western region with findings reported from only three districts. Hence, the findings might not be representative of the whole region and could not be generalized.

## Conclusion

This study has its merit in being the first study to be conducted in Uganda documenting PWDs informal support and needs. It revealed that various forms of informal support are available for PWDs in South Western Uganda and stressed the role of caregivers—be it family members, community helpers or leaders—in supporting PWDs in activities of daily living, assisting them in carrying out occupational activities, providing counseling services, creating a less isolated environments, offering herbal remedies, enabling them get to access to medical attention as well as achieving fulfilment, peace of mind and better quality of life with PWDs feeling loved, valued and appreciated. We noted that local custom encouraged the use of herbal remedies and felt they were helpful for PWDs. Even if it is only placebo effect, looking further into this information may yield culturally acceptable results. Having established the nature of informal support for PWD in rural south western Uganda, our findings can lay a foundation for future interventions on improving mental health care in families and communities at large especially for people with dementia in south western Uganda and other resource constraint settings. For all these findings and for much more, the authors urge for thorough research and available funding for this field to help understand inherent elements of dementia in Uganda and guide healthcare policies nationally and in Africa for people affected and living with dementia.

## Data Availability

The datasets used and analysed during the current study are available from the corresponding author on reasonable request.

## Appendix 1: Interview guide

Code: _______________________

I would like to appreciate your willingness to participate in this study and for allowing me time with you today.

I am NANKINGA NGOMA PIA undertaking a study on informal support for people with Alzheimer’s disease and related dementia in Southwestern Uganda. I would like you to share with me about the informal support you provide the person with dementia in this area. Dementia is a disease that comes with forgetfulness in old age. Feel free to share with me. The information you give me will be kept confidential and will only be used for purposes of this study. This interaction will take not more than an hour and to make it possible for me to get everything we shall talk about, I shall record this interaction. Kindly be audible enough so that I can capture our interaction without missing out any information.

General information

1. Gender of the participant.
2. Age of the participant.
3. Level of education of the participant.
4. Occupation of the participant.
5. How long have you been taking care of this person with forgetfulness?

I understand you are in direct contact and taking care of this person with forgetfulness

- What do you usually do for this person?
- How does this person benefit from what you do for him/her?
- Are there any other people that help this person while he/she is here in the home?

d) How and what do they usually do for this person?

e) How does this person benefit from what they do for him/her?

## Appendix 2: Translated interview guide

Code: ________________________

Ninyenda kusiima ahabw’okwikiriza kwejumba omu mushomo ogu hamwe n’okunyikiriza okampa obwiire bwaawe erizooba.

Ndi NANKINGA NGOMA PIA ndiyo ninkora ahamushomo gw’obuhagizi bwa buriijo ahabw’abantu abeine endwaaara y’okwekwata kwobwongo erikuguma nekura kandi erikweyongyera (Alzheimer’) hamwe nebikwatireine n’okwekwata kw’obwongo omuri Uganda eyamashuuma. Ninshaba onganiirire ahabikwatireine n’obuhagizi obwabureijo obu orikuhereza omuntu oyine okwekwata kwobwongo omu kyanga eki. Okwekwata kw’obwongo nendwaara erikwiija n’okwebwa yebwa omu myaaka ya bukuru. Ohurire otebekeine kuganiira nanye. Amakuru agu orikumpa nigeija kubiikwa nk’ekihama kandi nigeija kukozetsibwa ahabwomushomo ogu gwonka. Ekiganiiro eki tikirarenzye eshaaha emwe, kandi kubaasa kumbasisa kukwata buri kimwe eki turagambeho, ninyija kukwata ekiganiiro eki aharutambi. Noshabwa kugamba munonga kugira ngu mbaase kukwata ekiganiiro kyeitu ntafereirwe ‘makuru goona.

EBIKWATIREINE AHA AYAYEJUMBA OMU MUSHOMO

1. Omwojo ninga omwishikyi?
2. Emyaka y’omujumbi
3. Orurengo rw’okushoma
4. Nokorakyi kwebisaho
5. Wamara obwire burikwinganakyi orukureberera ogu muntu ayine obuzubu obu

Ninyetegyereza ngu ohikeine gye n’omuntu ogu oyine okwebwayebwa kandi nomureberera.

- Omuntu ogu nokira kumukoreraki?
- Omuntu ogu naganyirwamu ata omu bintu ebimurikumukorera?
- Hariho abandi bantu abarikuyamba omuntu ogu yaaba ari aha omuka?
- Omuntu ogu nibamukoreraki kandi nibakikora bata?
- Omuntu ogu naganyirwamu ata omu bintu ebi abantu aba barikumukorera?

## Appendix 3: Key informants guide

Code:_______________________

I would like to appreciate your willingness to participate in this study and for allowing me time with you today.

I am NANKINGA NGOMA PIA undertaking a study on informal support for people with Alzheimer’s disease and related dementia in Southwestern Uganda. I would like you to share with me about the informal support you provide the person with dementia in this area. Dementia is a disease that comes with forgetfulness in old age. Feel free to share with me. The information you give me will be kept confidential and will only be used for purposes of this study. This interaction will take not more than an hour and to make it possible for me to get everything we shall talk about, I shall record this interaction. Kindly be audible enough so that I can capture our interaction without missing out any information.

General information

1. Gender of the participant.
2. Age of the participant.
3. Level of education of the participant.
4. Occupation of the participant.

I understand there are people usually above the age of 60 with forgetfulness in your community.

- How common is it for people with this condition to seek for outside help?
- Is there any structure in the community to provide this help?
- How does this structure work? (probe: if there are individuals within the community designated to provide such support)
- What do you or the designated individual usually do for them in that situation?
- How helpful, do you think, what you do for these people is? (Probe: are there any notable changes?)
- Have you heard about “okushandaga eitaka? (probe: tell us about it)
- Under what circumstances such a practice would be done?

## Appendix 4: Translated key informants guide

Code: ________________________

Ninyenda kusiima ahabw’okwikiriza kwejumba omu mushomo ogu hamwe n’okunyikiriza okampa obwiire bwaawe erizooba.

Ndi NANKINGA NGOMA PIA ndiyo ninkora ahamushomo gw’obuhagizi bwa buriijo ahabw’abantu abeine endwaaara y’okwekwata kwobwongo erikuguma nekura kandi erikweyongyera (Alzheimer’) hamwe nebikwatireine n’okwekwata kwobwongo omuri Uganda eyamashuuma. Ninshaba onganiirire ahabikwatireine n’obuhagizi obwabureijo obu orikuhereza omuntu oyine okwekwata kwobwongo omu kyanga eki. Okwekwata kwobwongo nendwaara erikwiija n’okwebwa yebwa omu myaaka ya bukuru. Ohurire otebekeine kuganiira nanye. Amakuru agu orikumpa nigeija kubiikwa nk’ekihama kandi nigeija kukozetsibwa ahabwomushomo ogu gwonka. Ekiganiiro eki tikirarenzye eshaaha emwe, kandi kubaasa kumbasisa kukwata buri kimwe eki turagambeho, ninyija kukwata ekiganiiro eki aharutambi. Noshabwa kugamba munonga kugira ngu mbaase kukwata ekiganiiro kyeitu ntafereirwe ‘makuru goona.

EBIKWATIREINE AHA AYAYEJUMBA OMU MUSHOMO

1. Omwojo ninga omwishikyi?
2. Emyaka y’omujumbi
3. Orurengo rw’okushoma
4. Nokorakyi kwebisaho

Ninyetegyereza ngu hariho abantu abari ahiguru y’emyaaka 60 bureijo abeine okwebwa yebwa omu bantu b’omukyaaro kyanyu.

- Kikanyire kita abantu aba beine embeera egi kwiija kuronda obuyambi?
- Hariho entebekanisa yoona y’okuhereza obuyambi obu?
- Entebekanisa egi nekora eta?(Cumitiriza: kuhakuba harimu abantu abatoreinwe kuhereza obuhagizi nkobwo)
- Iwe nari ondeijo omuntu otoreinwe nimukira kukoreraki abantu abeine embeera nk’egyo?
- Noteekateeka ngu ebi orikukorera abantu aba nibibayamba bita?
- Wahurireho ebikwatirine nenkora eyokushandaga eitaka? (Tugambire aha ebyegyo enkora)
- Ninshonga kyi erikubaasa kuretaho egyo enkora eyokushanda eitaka?

## Abbreviations

PWD: People with dementia
VHTs: Village Health Team members
CSI-D: Brief Community Screening Interview for Dementia
